# Preclinical Studies on the Effect of Rucaparib in Ovarian Cancer: Impact of BRCA2 Status

**DOI:** 10.3390/cells10092434

**Published:** 2021-09-15

**Authors:** Sayeh Saravi, Zena Alizzi, Sabrina Tosi, Marcia Hall, Emmanouil Karteris

**Affiliations:** 1 Division of Biosciences, College of Health, Medicine and Life Sciences, Brunel University London, Uxbridge UB8 3PH, UK; sayeh.saravi@brunel.ac.uk (S.S.); zena.Alizzi@brunel.ac.uk (Z.A.); sabrina.tosi@brunel.ac.uk (S.T.); 2 Mount Vernon Cancer Centre, Rickmansworth Road, Northwood HA6 2RN, UK; 3 Division of Thoracic Surgery, The Royal Brompton & Harefield NHS Foundation Trust, Harefield Hospital, Harefield, Uxbridge UB9 6JH, UK

**Keywords:** ovarian cancer, PARP inhibitors, rucaparib, BRCA mutation, homologous recombination deficiency

## Abstract

Background: Approximately 50% of ovarian cancer patients harbour homologous recombination repair deficiencies. These deficiencies have been successfully targeted using poly (ADP-ribose) polymerase inhibitors (PARPi) particularly for patients harbouring BRCA1/2 mutations. The aim of this study is to assess the effects of the PARPi rucaparib in vitro using cell lines with BRCA2 mutations in comparison to those with BRCA2 wild type. Methods: Cell proliferation assays, RT-qPCR, immunofluorescence, annexin V/PI assays were used to assess the effects of rucaparib in vitro. Results: The BRCA2 mutant ovarian cancer cell line PEO1 exhibited higher PARP1 activity when treated with H_2_O_2_ compared to wild type cell lines. The migratory and proliferative capacity of PEO1 cells was compromised following treatment with rucaparib 10 µM compared to BRCA2 wild-type cell lines via a mechanism involving the mTOR pathway. Rucaparib treatment significantly increased DNA damage primarily in PEO1 cells and SKOV3 cells compared with wild type. Conclusions: Appropriate identification of robust predictive biomarkers for homologous recombination deficiency using ‘liquid’ biopsies would facilitate the identification of patients suitable for PARPi therapy. Preliminary efforts to undertake such testing are described here. This study also demonstrates the mechanisms of action of rucaparib (PARPi) which may involve elements of the mTOR pathway.

## 1. Introduction

Median progression-free survival for relapsed ovarian cancer (ROC) patients, who last had treatment 3–12 months previously, is 4–9 months with overall survival of ~12–20 months. There is an urgent need to improve outcomes for these patients [1,2]. Approximately 50% of ovarian cancer patients harbor homologous recombination repair deficiencies (HRD) [3]. HRD has been successfully exploited using poly (ADP-ribose) polymerase enzyme inhibitors (PARPi) in patients with germline BReast CAncer gene 1/2 (BRCA1/2) mutations (BRCAm) [4,5]. Of the two DNA damage PARP enzymes, PARP1 is responsible for protecting normal cells from toxic recombinogenic lesions occurring in damaged DNA. PARP1 enzyme plays a role as a first responder, detecting DNA damage and recruiting key proteins for repair. The inability to undertake homologous repair where cells lack BRCA1 or BRCA2, together with inhibition of PARP-1 results in an accumulation of DNA damage resulting in apoptosis [6]. However, ~80% patients with ROC are BRCA1/2 wildtype (BRCAwt), yet some of these patients also respond to PARPi albeit for shorter periods of time [7,8]. Extensive genome-wide loss of heterozygosity has been shown to identify BRCAwt ovarian cancer patients with HRD (also referred to as having “BRCAness”) who are more likely to respond to PARPi [9,10]. To date, there is a lack of accessible ‘liquid’ biomarkers to identify ovarian cancer patients harboring “BRCAness”.

Phosphorylated histone variant H2A, histone family member X (H2AX), i.e., γ-H2AX is very well described for its role in DNA damage repair following radiation and its use as a biomarker after chemotherapy is emerging [11]. Examination of γ-H2AX levels in normal tissue—plucked eyebrow hair follicles—from patients treated with a PARPi in a Phase 1 clinical trial showed a clear relationship between γ-H2AX levels, denoting double strand (ds) DNA damage, and PARP inhibition [12,13]. However, little is known about the expression and role of γ-H2Ax in circulating ovarian cancer-associated cells (CC) from blood (‘liquid biopsies’), although evidence of γ-H2AX expression exists in CC from Stage IV breast cancer patients undergoing platinum therapy [14]. The PARPi rucaparib has demonstrated clinical efficacy in ovarian cancer patients with BRCA1/2 (BRCAm) mutations and in BRCA wild type patients with homologous recombination deficiency (HRD) as indicated by a high degree of loss of heterozygosity [10].

The mechanistic target of rapamycin (mTOR) pathway is a crucial regulator for several pathways including growth, proliferation, apoptosis, and angiogenesis providing a balance between cellular resources, e.g., amino acids, growth factors, and stresses, such as hypoxia, to regulate behavior. It is emerging that the mechanistic target of rapamycin (mTOR) pathway may also be involved in DNA replication and genome stability [15,16]. Previous studies from our laboratory, have also shown the importance of this signaling pathway in ovarian cancer [17,18]. The expression of the mTOR complexes 1 and 2 (mTORC1, mTORC2) may change following treatment with rucaparib; assessment of the levels of the key accessory proteins, Raptor, Rictor and DEP domain containing mTOR interacting protein (DEPTOR) help to determine this.

The aim of this study is to assess the effects of rucaparib in vitro as a precursor to exploring the use of these techniques in vivo in patients with BRCA mutations and HRD or “BRCAness”. Moreover, we expand on our previous observations to study the effect of rucaparib, as an example of a PARPi, on the mTOR signaling pathway.

## 2. Materials and Methods

### 2.1. Cell Lines

PEO1 adherent ovarian cancer cell line was derived from high grade serous ovarian carcinoma (HGSOC) with a defect in the HR DNA double-strand break (DSB) repair pathway (i.e., BRCA2 mutation). PEO4 cells are an adherent cell line from the same patient as PEO1, but they have a reversion mutation of BRCA2 and are thus HRD proficient. SKOV3 are adherent and hypo-diploid cells, derived from a patient with ovarian cancer and considered to be a HGS cell line. MDAH-2774 cells are derived from patient with ovarian endometrioid carcinoma. Both SKOV3 and MDAH-2774 express BRCA2 wt (Table 1).

### 2.2. Cell Culture

The cell lines were cultured in T75 cell flasks with a filter head (Nunc) (Life Technologies Ltd, Paisley, UK), supplemented with 10% fetal bovine serum and 1% penicillin-streptomycin (Fisher Scientific, Leicestershire, UK). Cells were incubated at 37 °C in a humidified atmosphere of 5% CO_2_ in air. Cells were sub-cultured at 80% confluency, by trypsinization with TE (TrypLE Express, Gibco). Cell counts and viability were detected with “Countess™” (Fisher Scientific, Leicestershire, UK) automated cell counter utilizing the trypan blue exclusion method.

### 2.3. Cell Viability

The indicated dose of rucaparib was added in different concentrations: 10 µM for PEO1, PEO4 and MDAH-2774, and 25 µM for SKOV3 cell lines. By using an automatic cell counting chamber slide (Countess^®^ Automated Cell Counter, Invitrogen) with trypan blue, we assessed proliferation, death and viability of the cells after rucaparib treatment. At each time-point (24-h, 48-h and 72-h) media was aspirated, cells were trypsinized and re-suspended in 1 mL tissue culture media. An equal volume of cell suspension (10 μL) was mixed thoroughly with trypan blue and applied to a slide. An average value of three readings of cell numbers was calculated for each slide.

### 2.4. Annexin V/PI Assay

Using a 6-well plate, cells were treated in a dose- and time-dependent manner with DMSO (0.1%) and rucaparib, retaining an untreated control. FITC annexin V apoptosis detection kit with PI (BioLegend, San Diego, USA) was used for cell apoptosis analysis. After trypsinizing and centrifugation at 1500× *g* for 5 min, cells were washed twice with cell staining buffer and re-suspended in 100 µL Annexin V binding buffer. A total 5 µL of FITC Annexin V (488 nm) and 10 µL of propidium iodide solution (610 nm) were added and each tube incubated for 15 min at 25 °C in the dark. Compensation samples were made using unstained, untreated FITC stained, and untreated PI-stained cells. Next, 400 µL of Annexin V binding buffer was added and each sample analyzed by Flow Cytometry ACEA Novocyte Flow Cytometer. A total 12,000 cells were acquired for both treated and untreated samples; PI histograms were plotted using set markers within the analysis program of Novoexpress™ software.

### 2.5. PARP1 Activity Assay

Cells attached on poly-prep slides were incubated for 24-h at 37 °C. Following the addition of rucaparib or DMSO (0.1%) and further incubation for 1.5-h, PARP-1 activity was stimulated by treating with 20 mM H_2_O_2_ for 20 min at room temperature in the dark. Cells were fixed in 4% PFA for 10 min, then permeabilized with 0.5% Triton-X for 10 min before blocking with 5% BSA for 1-h at room temperature. Following incubation for 1-h, with a 1:200 dilution of mouse anti-PADPR monoclonal antibody (Abcam, Cambridge, UK), goat anti-mouse Alexa Fluor 488-conjugated secondary antibody (Abcam, Cambridge UK) was added, at a dilution of 1:500. Slides were triple washed for 5 min in TBST (Tris-buffered saline with Tween 20, PH7.5, (Merck Millipore, Dorset, UK) and then PBS and de-hydrated in ethanol series ((70%, 90% and 100%) for 3–5 min. A total 10 μL DAPI (Vector Laboratories, Burlingame, USA) was added to each air-dried slide and the cover slip sealed using clear nail varnish. Analysis was undertaken by Leica DM4000 microscopy. Fluorescence intensity for AF-488 was calculated by applying ImageJ software and normalizing to DAPI fluorescence intensity.

### 2.6. Blood Samples

Blood samples from BRCA mutant (*n* = 3) and BRCA wild type (*n* = 3) ovarian cancer patients were collected from Mount Vernon Cancer Centre, East and North Herts NHS Trust, as part of the CICATRIx study: Sample collection study to explore circulating tumor cells, cell free DNA and leucocytes with ImageStream analysis in patients with various cancers. Protocol number RD2016-08, approved by the West Midlands–South Birmingham Ethics Committee (reference 16/WM/0196) (Supplementary Table S1).

### 2.7. γ-H2AX Detection in Clinical Samples

All blood samples were collected in Roche tubes. Red cell lysis was carried out by incubating 1 mL of blood sample with 9 mL of RBC lysis buffer on a shaker for 10 min. Following centrifugation at 2500 RPM for 10 min, the supernatant was discarded and 3 mL of RBC lysis buffer was added to the cell pellet. It was incubated again on a shaker and re-centrifuged. This pellet was re-suspended in one mL PBS and cytospun for 5 min/800 rpm to attach the cells to slide. Slides were fixed in 4% PFA and cell membranes permeabilized with 0.5% Triton-X on ice. Following 1-h blocking, the -H2AX antibody conjugated Alexa Fluor (488) was added and the slides left overnight. Then the pellet was washed three times with 0.1% Tween in PBS for 5 min. After dehydrating with gradient ethanol (70%, 90% and 100%), 15 µL DAPI was added and slides were covered with a cover slip, sealed using clear nail varnish for analysis under the Leica DM4000 microscope. 100 cells were counted per slide and the number of cells positive for -H2AX documented.

### 2.8. Immunofluorescence—γ-H2AX In Vitro Assay

Cells were treated in dose- and time-dependent manner with DMSO (0.1%) and rucaparib. After washing in PBS, cells were fixed using 4% paraformaldehyde (PFA) in PBS for 15 min. Membrane permeabilization was undertaken using 0.2% Triton-X solution (Merck Millipore, Dorset, UK) in dH2O for 10 min at 4 °C, and non-specific sites blocked using 100 μL 2.5% bovine serum albumin (BSA) per slide. Slides were covered with parafilm and placed in a humidified dark box for one hour. Then, 100 μL of diluted γ-H2AX antibody conjugated with Alexa Fluor (Merck Millipore, Dorset, UK), at the relevant concentration (1:100) (following manufacturer’s instructions), was added to the slides. The slides were recovered with parafilm and placed in a humidified dark box for one hour. Slides were triple washed for 5 min in TBST (Tris-buffered saline with Tween 20, PH7.5, Merck Millipore, Dorset, UK) and then PBS. The slides were de-hydrated in gradient ethanol series for 5 min each time. Next, 15 μL DAPI (Vector Laboratories, Burlingame, USA) was added to each air-dried slide and the cover slip sealed using clear nail varnish. The slides were analyzed under a Leica DM4000 microscope.

### 2.9. RNA Isolation, cDNA Synthesis and Quantitative RT-PCR

Cells, plated in a 6-well plate, were treated in dose- and time-dependent manner with DMSO (0.1%) and rucaparib. RNA was extracted using the RNeasy Mini Kit (Qiagen, Manchester UK). cDNA was synthesized from mRNA utilizing cDNA reverse transcription (Life Technologies Ltd, Paisley, UK) cDNA concentration was controlled using RNA concentrations defined by Nano-Drop 2000C (Life Technologies Ltd, Paisley, UK). Relative expression of the genes of interest was measured by quantitative PCR (qPCR) on QPCR QuantStudio 7 Flex Real-Time PCR machine using SYBR^®^ Green PCR Master Mix (Life Technologies Ltd, Paisley, UK) using primers detailed in Table 2. RQ values were calculated according to the comparative 2^−ΔΔCq^ analysis method [19].

### 2.10. Statistical Analysis

Changes observed in experiments were assessed for statistical significance using the Student’s *t*-test and ANOVA (Analysis of Variance) test. All statistical tests were performed using GraphPad Prism^®^ (GraphPad Software). Values were considered as significant when *p* < 0.05.

## 3. Results

### 3.1. PARP1 and Cell Proliferation Assay

**Figure 1:**
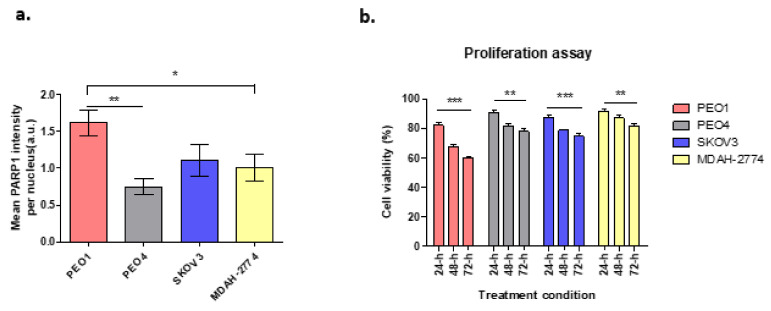
(**a**) PARP1 enzyme expression in cell lines treated with H_2_O_2_. PEO1: ** *p* = 0.0024, * *p* = 0.042; (**b**) Cell viability following rucaparib treatment at 24–72 h. PEO1 cells showed high level of decrease in cell viability after 72-h treatment when compared to other cell lines. However, the decrease level of cell viability in SKOV3 was still higher compared to PEO4 and MDAH-2774. Comparisons between 24 and 72-h of treatment; PEO1: *** *p* = 0.0002, PEO4: ** *p* = 0.0037, MDAH-2774: ** *p* = 0.0096, and SKOV3: *** *p* = 0.0003.

Cells with BRCA2 mutation have been shown to be highly sensitive to PARP1 inhibition which leads to apoptotic cell death due to the absence of BRCA-dependent HR repair, in contrast to those with efficient/wild-type BRCA2. Figure 1a demonstrates the expression of PARP1 enzyme following treatment with the DNA damaging agent hydrogen peroxide (H_2_O_2_) at 20 mM, in different ovarian cancer cell lines in terms of BRCA2 status. In comparison to untreated cells, there was a 28.8-fold increase in PARP1 expression in PEO1cells, which lack functional BRCA2. In contrast, PARP1 expression increased 5-fold for PEO4 cells, 12.5-fold for MDAH-2774 and 15.3-fold for SKOV3, all with functional BRCA2 (Figure 1a).

Based on previous clonogenic assays, 10 µM of rucaparib was chosen, based on the cytotoxicity reported in IC50 results [20], to investigate the effects of rucaparib in vitro. Cell viability was unaltered in cell lines treated with DMSO alone. Cells treated with rucaparib demonstrated a significant decrease in numbers of live cells over 24–48 h (Figure 1b). In BRCA2m cells (PEO1), rucaparib treatment resulted in significantly fewer live cells compared to other functional BRCA2 cell lines (Supplementary Figure S1).

### 3.2. Wound Healing Assay

**Figure 2:**
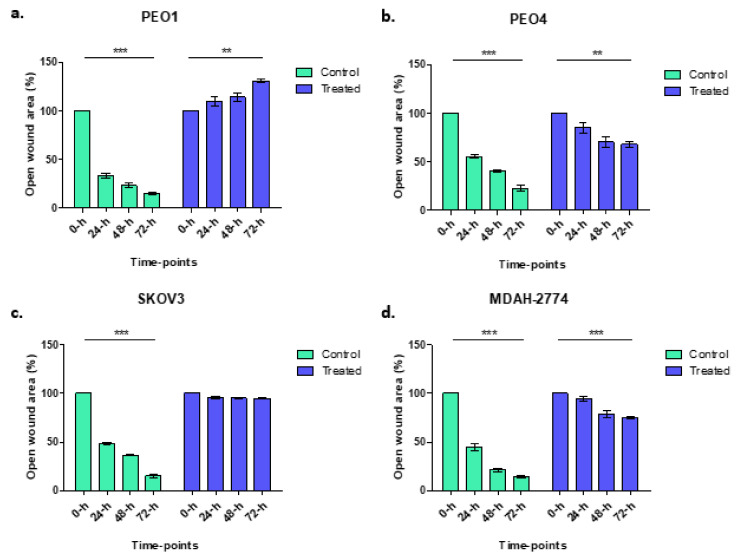
Control samples (green columns) from wound healing assays demonstrate gap closure where cells grow and migrate. Gap areas in the wound assays for all controls, irrespective of cell line, were significantly larger at 0 h than 72 h (*** *p* < 0.0001). There was no significant change in gap size after rucaparib treatment in SKOV3 cells (treated: *p* = 0.1088, BRCA2 wt, panel **c**). There was an increase in gap area following rucaparib treatment BRCA2m PEO1 cells (panel **a**) (** *p* = 0.0018). Rucaparib-treated PEO4 and MDAH-2774 cell lines (panels **b** and **d**, BRCA2 wt), demonstrated a reduction in open wound area although not as great as control samples (PEO4 ** *p* = 0.0059, MDAH-2774 *** *p* = 0.0006).

Wound healing assays were used to explore the migratory and proliferative capacity of ovarian cancer cells, in vitro, before and after treatment with rucaparib over 72 h. PEO4 and MDAH-2774 cells, both with functional BRCA2, demonstrated 60–75% gap closure, significantly less than untreated cells where 90% gap closure was seen; SKOV3 (BRCA2 wildtype) cells did not grow into the gap which remained identical. PEO1 (BRCA2m) cells, demonstrated an increasing gap width over time, presumably a consequence of a cytotoxic effect of rucaparib in BRCA mutant cells (Figure 2).

### 3.3. Rucaparib Induced Apoptosis in Ovarian Cancer Cells

**Figure 3:**
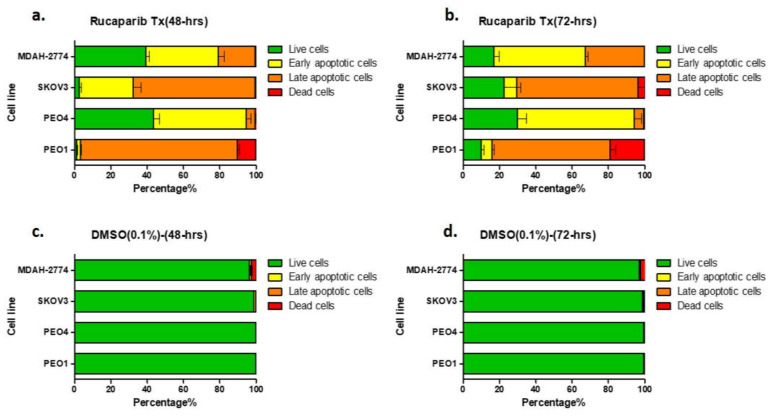
Annexin V assay. Proportion of cells demonstrating features of early apoptosis, late apoptotic cells, or no apoptotic features (live cells) in comparison to the proportion of dead cell, 48-h (**a**) and 72-h (**b**) after rucaparib treatment. Panels (**c**,**d**) are untreated controls.

There was a significant increase in the apoptotic population (early apoptosis; annexin V+/PI−, late apoptosis; annexin V+/PI+) as demonstrated by FACS, following treatment of all cell lines with 10 μM rucaparib (Figure 3, Supplementary Figure S1). 48-h following exposure to rucaparib, 10% PEO1 (BRCA2m) cells were dead and ~85% exhibited some features of apoptosis. The proportion of dead cells increased to 19% after a further 24 h (72-h post-treatment). In contrast, <1% PEO4 and MDAH-2774 cells (functional BRCA2) had died 72 h after treatment with rucaparib, although ~60% demonstrated features of apoptosis. Although significant numbers (95%) of SKOV3 cells (functional BRCA2) demonstrated apoptotic features 48 h following rucaparib, only ~5% were dead at 72 h and live cells with no features of apoptosis had increased from ~5% to >20% (Figure 3).

### 3.4. Rucaparib Treatment Significantly Increased DNA Damage

**Figure 4:**
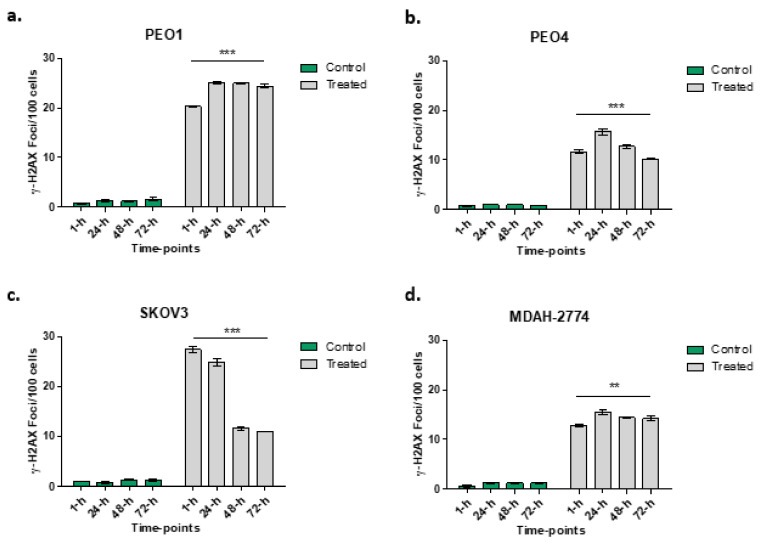
Numbers of γ-H2AX foci at different time points in PEO1 (panel **a**), PEO4 (panel **b**), SKOV3 (panel **c**) and MDAH-2774 (panel **d**) following rucaparib treatment. Comparisons between 1 h and 72-h of treatment undertaken: *** *p* < 0.0001, ** *p* = 0.0095.

An increased number of γ-H2AX foci in all cell lines following rucaparib treatment is demonstrated (Figure 4). More γ-H2AX foci, indicating greater DNA damage, were seen 1 h following treatment in PEO1 (27.6-fold) and SKOV3 (27.1-fold) cell lines than PEO4 (21.9-fold) and MDAH-2774 (20.3-fold). In both the PEO4 and MDAH-2774 lines, the numbers of γ-H2AX foci reduced to 12.6-fold and 11.8-fold respectively at 72 h from rucaparib. Amongst SKOV3 cells, the DNA damage appeared to increase initially, 32.2-fold at 24 h prior to a significant decrease in γ-H2AX by 72 h (8.9-fold). In PEO1 cells (BRCA2m), the increased number of γ-H2AX foci persisted up to 72 h (15.2-fold).

### 3.5. Effect of Rucaparib on mTORC1 and mTORC2 Components

**Figure 5:**
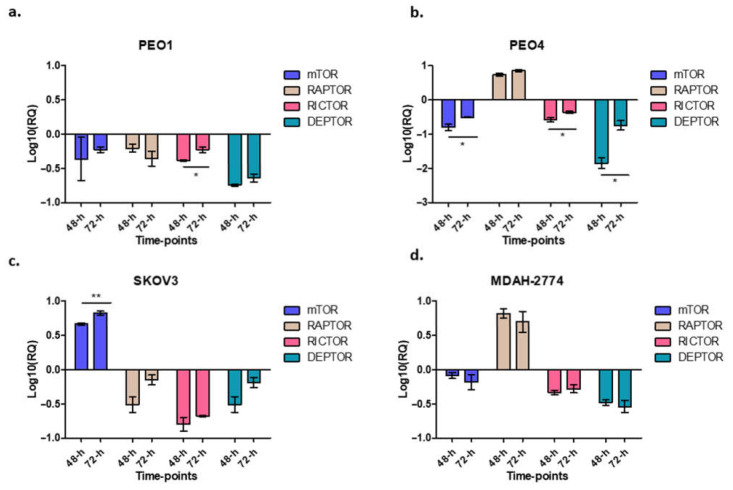
The figure represents the expression of different genes after rucaparib treatment in different time-points. Cells were treated with 10 μM rucaparib as single agents. Cells were harvested 48-h and 72-h after 10 μM rucaparib exposure. PEO1: RICTOR: * *p* = 0.0194 (panel **a**); PEO4: mTOR: * *p* = 0.0447, RICTOR: * *p* = 0.0322, DEPTOR: * *p* = 0.0136, (panel **b**); SKOV3: mTOR: ** *p* = 0.0092 (panel **c**), and no significant changes observed in MDAH-2774 (panel **d**).

In this study, when cells were treated with rucaparib (10 µM), it downregulated the expression of mTOR, raptor and rictor in PEO1 cells (Figure 5a), downregulated mTOR and rictor, but upregulated raptor in PEO4 and MDAH-274 (Figure 4d and Figure 5a) and upregulated mTOR, but downregulated rictor and raptor in SKOV3 cells (Figure 5c). A common feature for all cell lines was the downregulation of DEPTOR following rucaparib treatment.

### 3.6. Expression of γ-H2AX in Clinical Samples

**Figure 6:**
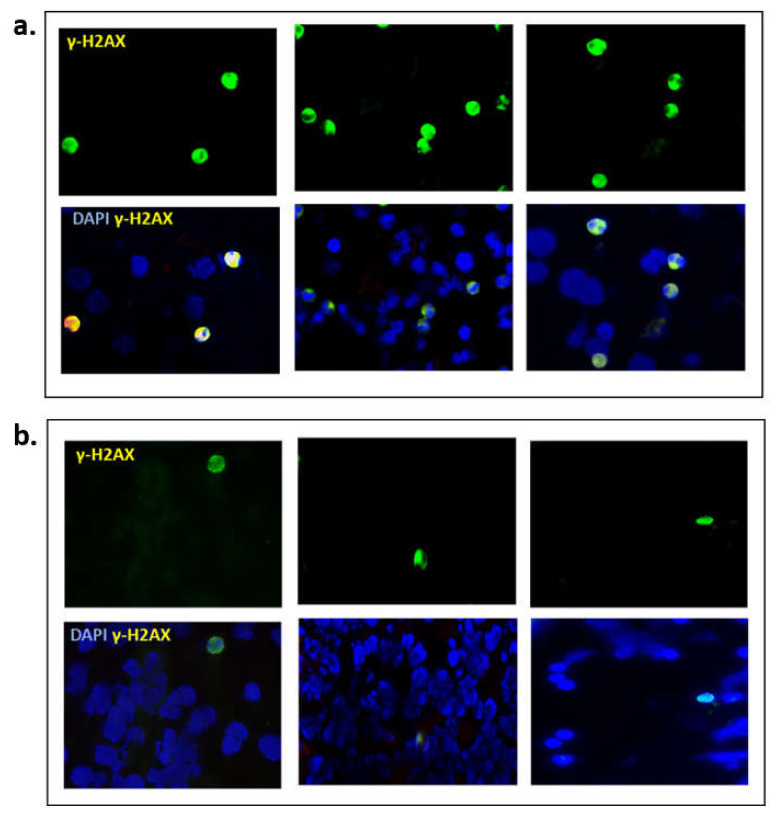
Representative images from (**a**) three BRCAm ovarian cancer patients (**b**) three BRCA wt patients, all undergoing chemotherapy. γ-H2AX positive cells were captured using immunofluorescence, magnification ×20.

Finally, we explored the clinical utility of γ-H2AX as a pharmacodynamic biomarker, in blood samples from BRCA mutant (*n* = 3) and BRCA wild type (*n* = 3) ovarian cancer patients. In this small cohort of enriched liquid biopsies, it was evident that BRCAm ovarian cancer patients undergoing chemotherapy had more positive γ-H2AX cells (mean 12.3/100 cells) compared with BRCA wt patients (mean 1.7/100 cells; Figure 6).

## 4. Discussion

Here we have explored the effects of rucaparib, an inhibitor of PARP in the context of BRCA2 mutation in ovarian cancer cell lines in vitro. We have confirmed the findings of Bryant et al., that inhibition of PARP1 enzyme is associated with a reduction in cell proliferation [21]. Not only does rucaparib exert the expected cytotoxic effect in cell lines with defective BRCA2 but we have demonstrated a reduced proliferative capacity for other cell lines with functional BRCA2. This reduction correlates with an overall increase in apoptosis and cell death in vitro, particularly in cell lines with defective BRCA2. As expected for a PARPi, rucaparib hinders DNA repair, resulting in phosphorylation of H2AX (γ-H2AX), a marker of double-strand DNA damage. It is interesting to note that both PE01 and SKOV3 cells had higher levels of γ-H2AX, implying difficulties in DNA repair. PE01 cells lack functional BRCA2, limiting their ability to undertake HR, which explains these γ-H2AX levels even at 72 h. SKOV3 cells have intact BRCA proteins, yet the initial surge in γ-H2AX suggests an element of HR deficiency, perhaps modelling “BRCAness”. The reduction in γ-H2AX after 24 h for SKOV3 suggests this effect is short-lived. Such findings have been correlated clinically where PARPi have been shown to have their greatest effect in patients with known BRCA mutations although a further ~30% patients with intact BRCA genes also benefit modestly, attributed to, as yet unknown, alternate HRD traits, resulting in “BRCAness” [22,23].

Furthermore, we assessed the expression of some of the genes involved in mTOR pathway, mTOR, rictor, raptor and DEPTOR, following treatment with rucaparib. As expected, PEO1 (BRCA2m) cells show a decrease in expression of, mTOR, raptor and rictor. This reflects the demonstrated reduction in growth and proliferation, metabolism and survival for these BRCAm cells after treatment with rucaparib. Similarly, there was a decrease in raptor and rictor expression in SKOV3 cells which also demonstrated short-lived BRCAness following treatment with rucaparib. Interestingly the raptor levels had almost recovered by 72 h. In contrast, PEO4 and MDAH-2774 (functional BRCA2) presented an increase in raptor expression correlating with the significantly lower effect of rucaparib in these cell lines. It raises the possibility that mTORC1/mTORC2 complexes may have a role in the increasing apoptosis seen after PARP inhibitor therapy (rucaparib) in HRD and BRCA2m cell lines, as we have suggested previously [24]. Rucaparib induced a downregulation of rictor in all cell lines, regardless of HRD/BRCA status, possibly indicating that PARPi can compromise cell mobility/viability via mTORC2 directly. Of note, a number of cancers have documented upregulation of rictor including lung cancer, sarcoma, neuroendocrine prostate, esophagus and stomach cancers, rendering this protein as a potential therapeutic target [25]. Additionally, in glioblastoma multiforme (GBM), epidermal growth factor receptor (EGFR) and rictor-mediated pathways have been shown to play a key role in chemoresistance (increased viability) [26]. The combination of everolimus (mTOR inhibitor) and olaparib (PARPi) has been tested in BRCA2-mutated breast cancer patient-derived xenografts (PDX) with promising results. This too is suggestive of a potential convergence of PARP and mTOR signaling pathways [6].

DEPTOR, a key regulator of both mTORC1 and mTORC2 complexes, was also downregulated in all four cell lines treated with rucaparib, reaching statistical significance in the PEO4 cell line. To date, its precise role in malignancies is still controversial, as it appears to acquire a dual-role, acting as a tumor suppressor in some cancers and as an oncogene in others. Studies have shown that although DEPTOR inhibits mTORC1, it can also activate the Akt pathway (by phosphorylation at S437 and T308 residues) to induce cell proliferation [27]. For example, in cervical squamous cell carcinoma cells DEPTOR induced cell survival, and overexpression of DEPTOR correlated with poor prognosis in differentiated thyroid carcinoma [28,29,30]. Yet, when silenced, DEPTOR promotes apoptosis via downregulation of PI3K/AKT pathway [27]. We, and others, have also shown that DEPTOR is overexpressed in chemotherapy-resistant ovarian cancer cell lines [31,32]. Future studies are planned to focus on assessing the effect at protein level as well as measuring changes in the phosphorylation status of key signaling molecules like Akt, mTOR and S6K.

Finally, the identification of robust predictive biomarkers for deficiencies in the homologous recombination pathway in cancer cells is a critically important challenge clinically [33]. Here we provide very preliminary evidence of the potential utility of measuring the quantities of γ-H2AX positive CCs as a predictive biomarker of response to PARP inhibitors clinically. Two previous studies corroborate these findings, in cancer patients [14,34]. Wang et al., demonstrated a 36% increase in γ-H2AX-positive CTCs from patients with a variety of different solid tumor malignancies (breast, colorectal, small cell lung and prostate cancers) enrolled in a variety of Phase I clinical trials of chemotherapy with and without PARP inhibitors. This increase occurred one day after treatment, irrespective of changes in the numbers of CCs [34].

## 5. Conclusions

In conclusion, we have confirmed that PARP1 is essential for HR in ovarian cancer cell lines lacking BRCA2 and treatment with rucaparib, a PARP inhibitor, increases the formation of γ-H2AX foci. In line with this, we have suggested that there are an increased number of γ-H2AX foci amongst circulating cells in patients harboring BRCA mutations compared with those with wild type BRCA. This finding requires validation but could be a key factor in identifying a more reliable marker of HRD in a clinical population. The reduction in expression of components of the mTOR complexes following PARP inhibitor therapy, corroborates proposals that PI3K/AKT/mTOR is also involved in DNA repair/replication in some settings. More work is needed to understand the interaction of these important cellular pathways and identify potential areas for therapeutic exploitation.

## Figures and Tables

**Table 1:** List of all cell lines used in this study. TP53: tumor protein 53, NF1: Neurofibromin 1, PIK3CA: Phosphatidylinositol-4,5-Bisphosphate 3-Kinase Catalytic Subunit Alpha, HRAS: HRas Proto-Oncogene, GTPase, ARID1A: AT-Rich Interaction Domain 1A, ERBB2: Erb-B2 Receptor Tyrosine Kinase 2, and KRAS: KRAS Proto-Oncogene, GTPase.

Cell Line	History	Mutations	Pathology
PEO1	Derived from ascites	*TP53*, chemosensitive *BRCA2*-mutant	HGSOC
PEO4	*BRCA2*-proficient line acquired by reversion mutation from PEO1 line following chemotherapy	*TP53*, *BRCA2* (silent)	HGSOC
SKOV3	Derived from ascites	*TP53*, *NF1*, *PIK3CA*, *HRAS*, *ARID1A*, *ERBB2*	Thought to be HGSOC
MDAH-2774	Derived from ascites	*TP53*, *KRAS*, *PIK3CA*, *ARID1A*, *BRCA1/2* (silent)	Endometrioid

**Table 2:** List of primers used for RT-qPCR in this study.

Gene name	Orientation	Sequence
YWHAZ	Forward	5′-AGACGGAAGGTGCTGAGAAA-3′
	Reverse	5′-GAAGCATTGGGGATCAAGAA-3′
mTOR	Forward	5′-TGCCAACTACCTTCGGAACC-3′
	Reverse	5′-GCTCGCTTCACCTCAATTC-3′
DEPTOR	Forward	5′-CACCATGTGTGTATGAGCA-3′
	Reverse	5′-TGAAGGTGCGCGCTCATTG-3′
Rictor	Forward	5′-GGAAGCCTGTTGATGGTGAT-3′
	Reverse	5′-GGCAGCCTTTTTATGGTGT-3′
Raptor	Forward	5′-ACTGATGGAGTCCGAATGC-3′
	Reverse	5′-TCATCCGATCCTTCATC-3′

## Data Availability

Data is available, upon reasonable request.

## Supplementary Materials

The following are available online at https://www.mdpi.com/article/10.3390/cells10092434/s1, Table S1: Patient characteristics. NACT: neo-adjuvant chemotherapy, Figure S1: Flow cytometry analysis with annexin V and propidium iodide staining in PEO1, PEO4, MDAH-2774 and SKOV3 cells showed a significant increase of apoptotic population (early apoptosis; annexin V+/PI−, late apoptosis; annexin V+/PI+) with treatment of 10 μM rucaparib. Flow cytometry analysis was performed 48-h and 72-h after treatment of rucaparib.
