# Supplementary material for: Preclinical Studies on the Effect of Rucaparib in Ovarian Cancer: Impact of BRCA2 Status
Source: Cells. 2021 Sep 15;10(9):2434. doi: 10.3390/cells10092434 (PMC8472031; doi:10.3390/cells10092434)
Supplement: Supplementary file 1 [file cells-10-02434-s001.zip › cells-1338849-supplementary.pdf]

Table S1. Patient characteristics. NACT: neo-adjuvant chemotherapy.

BRCA status	Age (years)	Treatment	γ-H2AX /100 cells
Mutant	77	NACT with surgery	21
Mutant	54	NACT with surgery	10
Mutant	52	Chemotherapy/Cisplatin	6
Wild type	62	NACT with surgery	2
Wild type	85	NACT with surgery	2
Wild Type	53	NACT with surgery	1

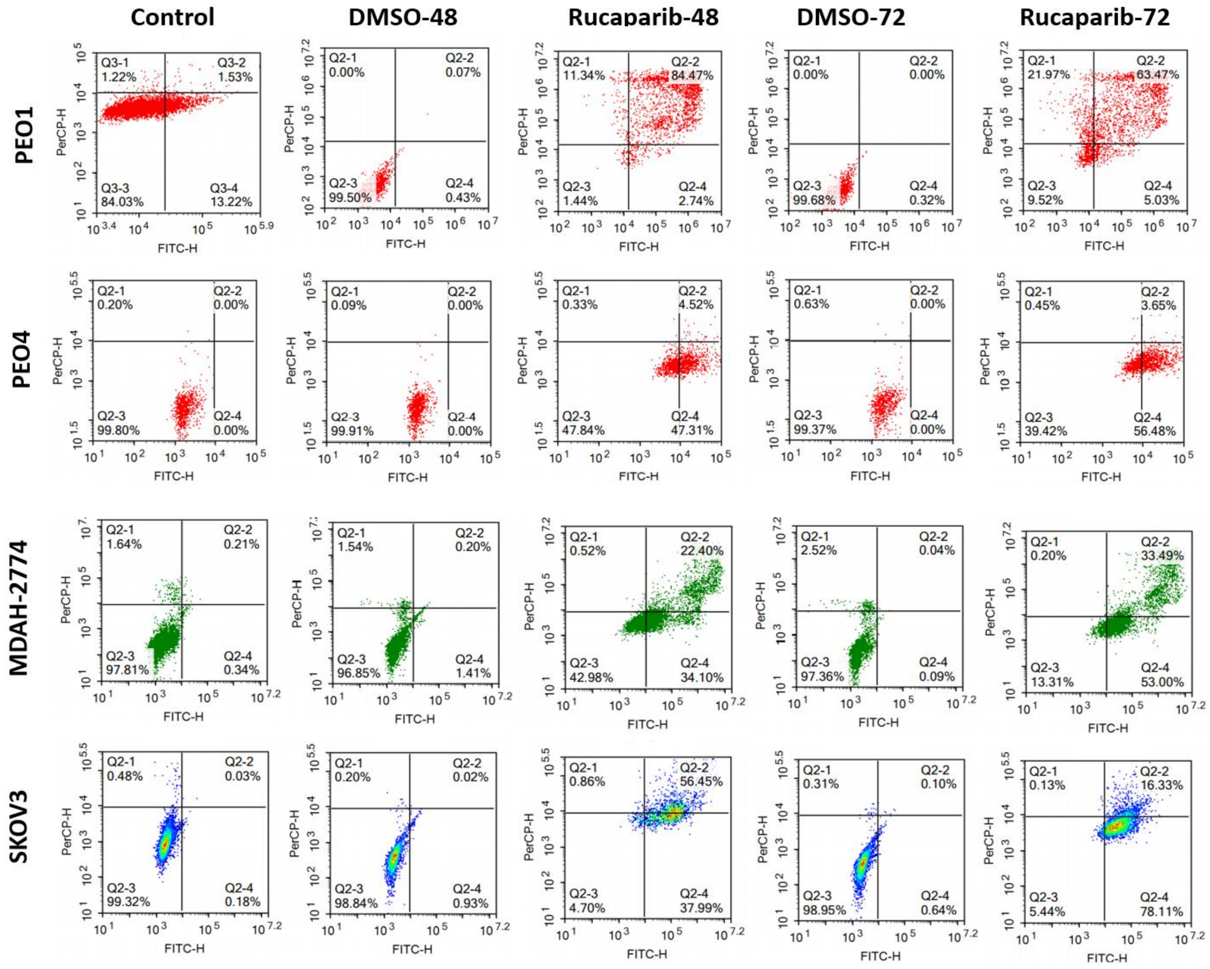

Figure S1. Flow cytometry analysis with annexin V and propidium iodide staining in PEO1, PEO4, MDAH-2774 and SKOV3 cells showed a significant increase of apoptotic population (early apoptosis; annexin V+/PI−, late apoptosis; annexin V+/PI+) with treatment of 10 μM rucaparib. Flow cytometry analysis was performed 48-hrs and 72-hrs after treatment of rucaparib.
